# Multi-character perspectives on the evolution of intraspecific differentiation in a neotropical hylid frog

**DOI:** 10.1186/1471-2148-6-23

**Published:** 2006-03-15

**Authors:** Stephen C Lougheed, James D Austin, James P Bogart, Peter T Boag, Andrew A Chek

**Affiliations:** 1 Department of Biology, Queen's University, Kingston, Ontario, Canada, K7L 3N6; 2 Departments of Wildlife Ecology & Conservation and Fisheries & Aquatic Sciences (IFAS), University of Florida, Gainesville, FL, USA, 32611; 3 Department of Integrative Biology, University of Guelph, Guelph, Ontario, Canada; 4 Organization for Tropical Studies, Box 90630, Durham, NC, USA

## Abstract

**Background:**

Multi-character empirical studies are important contributions to our understanding of the process of speciation. The relatively conserved morphology of, and importance of the mate recognition system in anurans, combined with phylogenetic tools, provide an opportunity to address predictions about the relative role of each in the process of speciation. We examine the relationship among patterns of variation in morphology, call characters, and 16S gene sequences across seven populations of a neotropical hylid frog, *Hyla leucophyllata*, to infer their relative importance in predicting the early stages of population differentiation.

**Results:**

Multivariate analyses demonstrate that both morphological and call characteristics were significantly variable among populations, characterized by significantly lower intra-population dispersion in call space than morphological space, and significantly greater among-population variation in call structure. We found lack of concordance between a 16S DNA phylogeny of *Hyla leucophyllata *and the significant population-level differentiation evident in both external morphology and male advertisement call. Comparisons of the reconstructed gene trees to simulated lineages support the notion that variation in call cannot be simply explained by population history.

**Conclusion:**

Discordance among traits may reflect sampling biases (e.g. single genetic marker effects), or imply a decoupling of evolution of different suites of characters. Diagnostic differences among populations in call structure possibly reflect local selection pressures presented by different heterospecific calling assemblages and may serve as a precursor of species-wide differentiation. Differentiation among populations in morphology may be due to ecophenotypic variation or to diversifying selection on body size directly, or on frequency attributes of calls (mediated by female choice) that show a strong relationship to body size.

## Background

The process of geographic speciation may be represented as the shift from panmixia, through polyphyly and paraphyly, to reciprocal monophyly of newly emerged sister species [1]. From this vantage, understanding speciation requires the study of historical and geographical factors that may underlie origins and diversification of lineages within species (e.g. isolation by distance, vicariance), and also the changes that occur in suites of characters that can affect survivorship or are important in reproductive isolation (e.g. morphology, mate recognition system). Multi-character phylogeographic perspectives are particularly fruitful in this regard, where the evolutionary history inferred from DNA sequence data provides a baseline for evaluating the divergence of various phenotypic attributes. In other words, evolutionarily independent, reciprocally-monophyletic lineages in similar environments can theoretically diverge in heritable phenotypic attributes due to genetic drift, with greater divergence predicted for more deeply diverged lineages simply because they have been separated for longer periods of time. Deviation from such expectation implies the action of selection or constraint, indicating that on some level the evolution of phenotype is decoupled from history.

Anurans (frogs) provide excellent systems for multi-character phylogeographic approaches. They are typically less vagile than other vertebrates (e.g. mammals and birds), presumably promoting the development of differentiation among populations and regions. Indeed, many tropical and temperate amphibian species exhibit striking phylogeographic structure and deep genetic divisions [e.g. [2,3], implying that significant spans of time have elapsed over which phenotypic differences could evolve. Many frog species breed in aggregations facilitating point sampling for geographic surveys. Finally frogs have a well-studied mate recognition system (hereafter – MRS), with a well-understood neuroethological basis and variation that is readily quantified and manipulated to test for its significance [4].

MRS was historically viewed as preventing fitness costs associated with inter-specific mating [5]. Frogs and their male-delivered advertisement calls have featured prominently in research illustrating such classical notions of MRS evolution (e.g. [6-8]. Other explanations for MRS evolution recently have gained greater acceptance focusing on (i) a role for sexual selection, especially through female preference for certain male traits [reviewed in [9]], or (ii) on direct selection on the MRS through predation, competition, and environmental effects (e.g. selection for signal transmission in different media) [10]. These hypotheses share the prediction that MRS evolution may precede the evolution of other hallmarks or correlates of species status (including morphological distinctiveness) and may even initiate speciation [11].

In this study we examined patterns of variation in morphology, phylogeny, and male advertisement call across "populations" of a widely distributed neotropical frog, *Hyla leucophyllata *[12]. Morphology tends to be conserved in frogs both within and among species (e.g. [13,14]), and it is distinction in the MRS that is the hallmark of speciation [4,15]. For this reason alone we might predict that the latter will exhibit greater differentiation among populations. However, we can embed predictions of among population divergence within a more formal theoretical framework. For example, in contrast to morphology, the MRS in frogs may be under intense stabilizing selection because of the fitness costs of inappropriate mate choice [e.g. [16]]. Thus, we might expect there to be little range wide variation in call at least in those characters important in female choice. Alternatively, differences in call characteristics among populations may mirror phylogeny if drift alone results in divergence. Further, call characteristics may not map onto phylogeny because the former diverges before differences in neutral characters accrue. In the present study we test: (i) if there are significant differences in call and morphology among seven populations of *H. leucophyllata*, (ii) if differentiation between populations in these two suites of traits are significantly correlated, and how each relates to spatial separation between populations (a potential predictor of both gene flow and degree of environmental similarity), and (iii) if divergence in phenotype, and particularly MRS, shows a significant relationship with genealogical history.

## Results

### Variation

**Figure 1:**
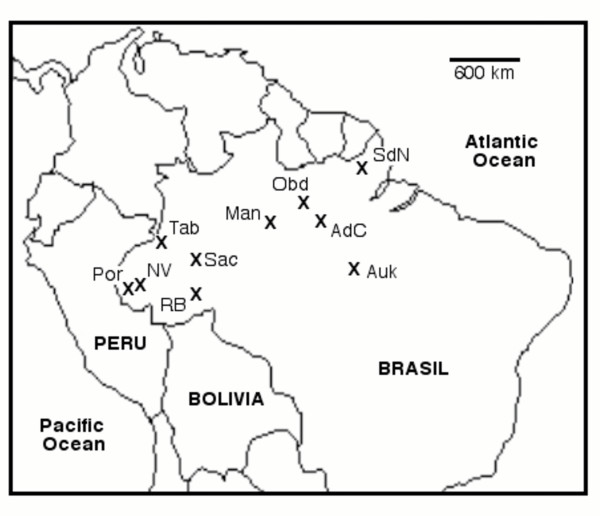
**Map of sampling locations. ***Locations from which *Hyla leucophyllata *and *H. triangulum *were sampled. AdC = Alter do Chão, Auk = A-Ukre, Man = Manaus, Obd = Obidos, RB = Rio Branco, SdN = Serra do Navio, Tab = Tabatinga, Por = Igarapé Porongaba, NV = Nova Vida, Sac *= Sacado.

We obtained between 490 and 503 base pairs for all 65 surveyed *H. leucophyllata *(see Figure 1 for locations), three *H. triangulum*, and two outgroup *H. elegans*. The 24 distinct ingroup haplotypes differed at between 1 and 46 sites (sequence divergence between 0.2 and 8.8 %, respectively), with an average ti/tv ratio, excluding comparisons with an undefined quotient, of 2.73. Average base composition was as follows: 25.3% A, 20.9% C, 24.4% G, and 29.3% T.

Coefficients of variation for morphology variables among populations (Additional file 2) ranged from 5.4 % (snout length) to 11.1 % (hand disc diameter). The first two axes of the CVA accounted for 77% (CV I = 56.7%, CV II = 20.3%) of the total morphological variance. All variables loaded positively on CV I (Additional file 3), but were not equal in magnitude suggesting that these axes represented some element of both size and shape [17]. Loadings on CV II were almost all negative, and here again magnitudes were variable suggesting some shape dimension in addition to size.

Coefficients of variation varied more widely for call variables than those for morphology varying from 2.2% (secondary pulse duty cycle) to 143% (secondary note FM sweep) (Additional file 4). Five characters varied significantly with body size (Table 3). The CVA of call variables accounted for 77% of among-population variance over the first two axes (CV I = 53.7%, CV = II 23.2%) (Additional file 5). A few temporally-based variables showed the heaviest loadings on CVA axes; generally, shorter primary notes with fewer pulses corresponded to greater pulse rates and number of secondary notes.

**Table 1:** Sample sizes of Hyla leucophyllata for each character set. For acronyms see Figure 1 legend.

**Sampling Location**	**DNA Sequence**	**Morphology**	**Call**
AdC	9	8	7
Auk	10	27	10
Man	10	11	3
Obd	12	17	12
RB	3	3	3
SdN	12	25	8
Tab	7	11	8
Por	1	-	-
NV	1	-	-

**Table 2:** Definitions of call variables measured for each Hyla leucophyllata call.

**Variable**	**Description**
Prim. Dom. Frequency	Frequency containing the most energy over the length of the primary note
Prim. FM Range	Dominant frequency difference between the beginning and end of the primary note
Prim. FM Sweep	Primary FM range divided by the primary note length
Sec. Dom. Frequency	Frequency containing the most energy over the length of the first secondary note
Sec. FM Range	Dominant frequency difference between the beginning and end of the first secondary note
Sec. FM Sweep	Secondary FM range divided by the first secondary note length
Call Length	Total length of call including all secondary notes
Inter-note Interval	Time between the end of the primary note and beginning of the first secondary note
Prim. Note Length	Length of primary note
Prim. Note & Inter-Note Inter.	Length of primary note plus the inter-note interval
Prim. Note Rise	Time from the beginning of the primary note until the maximum amplitude of the primary note is reached
Prim. Note Shape	Primary note rise time divided by primary note length
Number of Prim. Pulses	Number of pulses contained in the primary note
Prim. Pulse Length	Length of first clearly discernible pulse of the primary note
Prim. Pulse + Inter-pulse Inter.	Primary pulse length plus the time to the onset of the next pulse
Prim. Pulse Rise	Time from the beginning of first clearly discernible pulse of the primary note to that pulse's maximum amplitude
Prim. Pulse Shape	Primary pulse rise time divided by primary pulse length
Prim. Pulse Duty	Primary pulse length divided by primary pulse +inter-pulse interval
Prim. Pulse Rate	Number of primary pulses divided by the primary note length
Sec. Note Length	Length of first secondary note
Sec. Note Rise	Time from the beginning of the first secondary note until the maximum amplitude of the first secondary note is reached
Sec. Note Shape	Secondary note rise time divided by secondary note length
Number of Sec. Notes	Number of notes following the primary note
Number of Sec. Pulses	Number of pulses contained in the first secondary note
Sec. Pulse Length	Length of first clearly discernible pulse of the first secondary note
Sec. Pulse + Inter-pulse Inter.	Secondary pulse length plus the time to the onset of the next pulse
Sec. Pulse Rise	Time from the beginning of first clearly discernible pulse of the secondary note to that pulse's maximum amplitude
Sec. Pulse Shape	Secondary pulse rise time divided by secondary pulse length
Sec. Pulse Duty	Secondary pulse length divided by secondary pulse +inter-pulse interval
Sec. Pulse Rate	Number of secondary pulses divided by the secondary note length

**Table 3:** Regression statistics for call variables that showed a significant relationship to snout-vent-length (SVL). Of the tests for a relationship between SVL and other call variables, almost all had *p*-values between 0.3 and 0.8. Adjusted *p *refers to sequential Bonferroni correction for multiple tests [54]. Only the variables marked with an asterisk showed significant variation with SVL after correction. For these variables we used residuals in our CVA.

**Variable**	**r**^2^	**df**	**F**	***p***	**Adjusted *p***
2°dom freq*	0.676	1,45	94.14	<0.0001	0.0016
1°dom freq*	0.611	1,47	73.94	<0.0001	0.0017
1°prim pulses*	0.278	1,47	18.11	0.0001	0.0017
1°note + inter*	0.225	1,45	13.07	0.0008	0.0018
2°note rise*	0.211	1,45	12.09	0.0011	0.0019
2°note shape	0.183	1,45	10.09	0.0027	0.0020
1°length	0.128	1,47	6.92	0.0115	0.0020

### Population distinction in morphology and advertisement call

**Figure 2:**
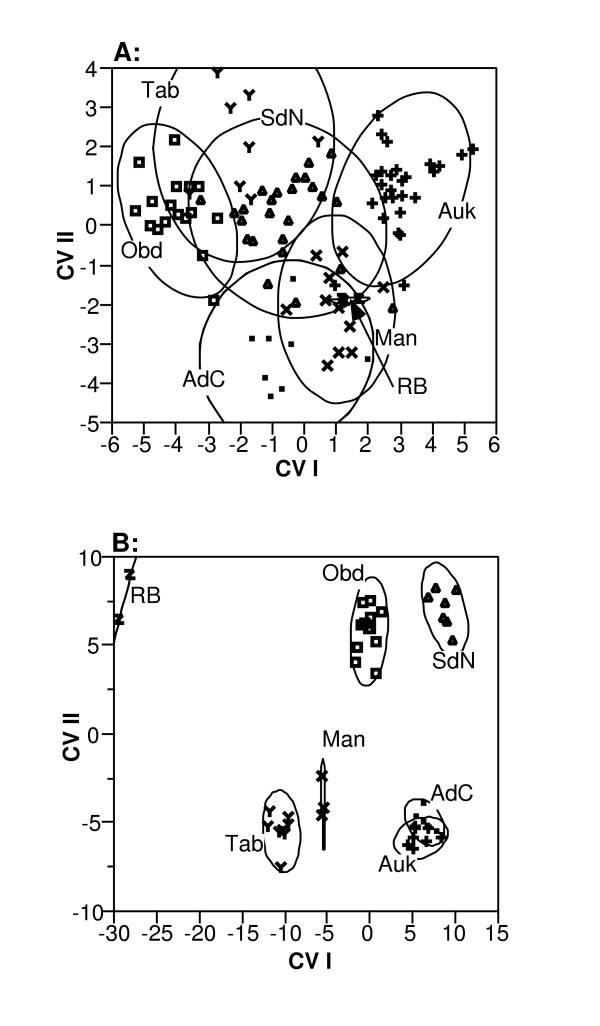
**Bivariate CVA plots for morphology and call. **Individual scores of *Hyla leucophyllata *individuals on first two canonical variates axes with 95% density ellipses shown for each population. Population codes follow Figure 1. **A: **morphology **B: **calls

All morphological variables differed significantly among populations, as indicated by highly significant Kruskal-Wallis tests (Additional file 2). However, separation among populations in the space defined by the first two CV axes was low relative to within-population scatter (Figure 2A). Nonetheless, *post-hoc *classification by the overall CV function was quite accurate (94.7%; Table 4) indicating diagnostic morphological variation among populations.

**Table 4:** Accuracy of CVA of the morphological data set in the post-hoc prediction of populations membership of Hyla leucophyllata individuals.

	**Observed population membership**						
	
**Predicted pop.**	AdC	Aukre	Man	Obd	RB	SdN	Tab
AdC	**7**	0	0	0	0	1	0
Aukre	0	**26**	0	0	0	1	0
Man	0	1	**10**	0	0	1	0
Obd	0	0	0	**17**	0	0	0
RB	1	0	1	0	**3**	0	0
SdN	0	0	0	0	0	**22**	0
Tab	0	0	0	0	0	0	**8**

**% corrrect**	87.5	96.3	90.9	100	100	88	100

After sequential Bonferroni adjustment, only 10 of the 30 call variables showed significant inter-population variation as judged by Kruskal-Wallis tests (Additional file 4). Of these 10 variables, five represented the heaviest loading factors on CV I. Separation along CV I and II was much more obvious in call than in morphological space (Figure 2B), a fact reflected by the increase in *post-hoc *classification success (100%) by the CV call function (data not shown). The increased discriminatory power of the call CV function is likely due to comparatively low intra-population dispersion in call space. A difference in intra-population dispersion is reflected in two explicit comparisons. First, when call and morphology CV matrices are scaled equivalently, median intra-population dispersion in call space (diagonal matrix elements) is indeed significantly lower than that for morphological space (Mann-Whitney 2 Sample, Z_approx_. = -3.066, n_1 _= 7, n_2 _= 7, p = 0.0022). Second, there is a difference in the coefficients of variation: the ten significantly different call variables had a higher average among-population coefficient of variation (28.3 ± 16.2 %) than morphological variables (8.6 ± 1.6 %); a difference that is highly significant (Mann-Whitney 2 Sample, Z_approx_. = 4.24, n_1 _= 10, n_2 _= 17, p < 0.0001).

### Comparison of morphology, call and geographic distance

Apportioning out the known affects of body size (SVL) on some call parameters (Table 3) in our call CVA, we found no relation between level of inter-population differentiation in call and morphology (Table 5). This was true using both ranked and unranked matrices. Distinctiveness in morphology between populations estimated from a CVA showed no relation to geographic distance; however, differentiation in advertisement call structure did show a clear and significant relationship (Table 5).

**Table 5:** Summary of pairwise Mantel's tests on inter-populational distance matrices. Matrices used in each test were derived from CVA of call and morphological variables, and from straight-line geographic distances between collecting localities. Top number in each cell is *r *(correlation coefficient), bottom is *p*-value. Comparisons marked by an asterisk were significant after sequential Bonferroni adjustment. Each matrix uses ranked values but the results are unchanged with raw (unranked) values

	**Call**	**Geog**
**Morph**	-0.1286 **0.3223**	-0.0104 **0.4824**
**Call**		0.6753 *0.0028

### Phylogenetics

**Figure 3:**
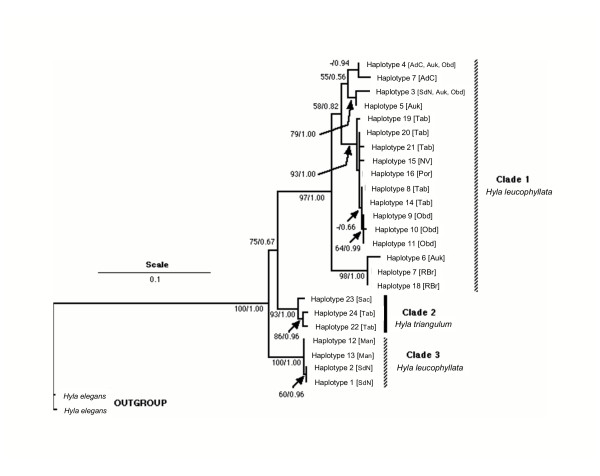
**Phylogenetic hypothesis derived from maximum likelihood analysis of 16S DNA sequence. **ML tree showing relationship among *Hyla leucophyllata *and *H. triangulum *haplotypes. Bootstrap support for ML and Bayesian posterior probabilities (before and after the forward slash) are indicated where the former exceeds 50% and the latter 0.70.

Topologies of trees from our maximum likelihood and Bayesian analyses were identical in almost all aspects and we present only the former in Figure 3. Both approaches showed three major well supported clades with > 90% bootstrap support (maximum likelihood) and posterior probabilities of 1.00 (Bayesian). One clade was comprised of three haplotypes found exclusively in *H. triangulum *(Clade 2 – Figure 3), while the other two contained only *Hyla leucophyllata *haplotypes. Clade 1 is distributed across the entire sampled range of *H. leucophyllata *with some well-supported phylogenetic structure within. Clade 3 is comprised of only four *H. leucophyllata *haplotypes from two sites (SdN and Man – see Figures 1 and 3). Two haplotypes (C and D) are shared among three eastern sites, and one sampling locale (SdN) contains haplotypes (C, B and A) from Clades 1 and 3. Pairwise divergence among haplotypes within clades ranged from 0.4% to 6.5% in Clade 1, 1.4% to 2.5% in Clade 2, and 0.1 to 0.2% for Clade 3. Point estimates of divergence among clades were 8.3%, 9.3% and 6.2% for Clade 1 verses Clade 2, Clade 1 verses 3, and Clade 2 verses Clade 3, respectively.

**Figure 4:**
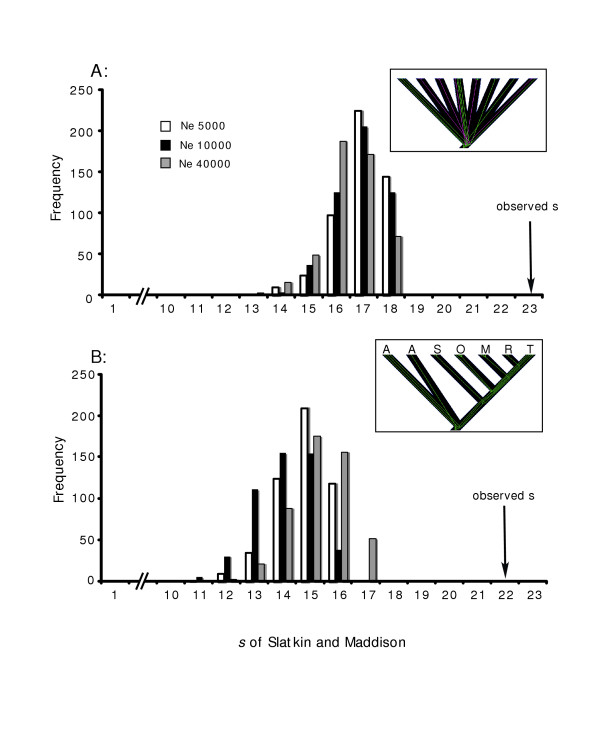
**Results of coalescent simulations comparing gene versus population histories. **Distributions of expected values of the test statistic, *s*, derived from 500 coalescent simulations over a variety of demographic histories. **A: **Eight populations originating from a common ancestral population representing a fragmentation model (inset). **B: **Seven populations structured by a matrix of distances from the CVA of calls, representing an isolation by (call) distance model (first letter of population indicates position on tree). Observed discord between gene tree and population tree is indicated with an arrow.

**Figure 5:**
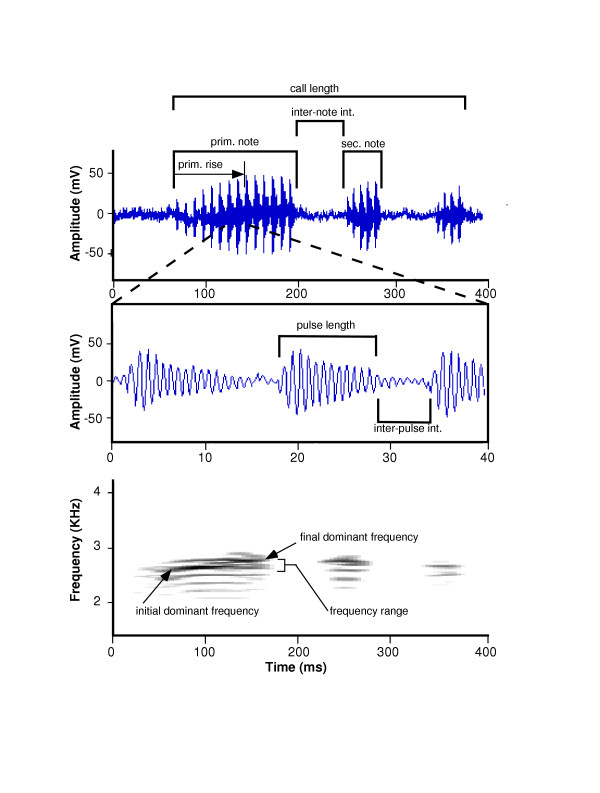
**Representative waveform and sonogram for *Hyla leucophyllata call *A**: waveform typical of *Hyla leucophyllata *male advertisement call showing primary note with two secondary notes **B**: expanded view of three primary pulses **C**: sonogram of call in (A).

Although highly structured phylogenetically, population history is complicated by the geographic distribution of deeply divergent, paraphyletic clades. The discordance was significantly greater for the reconstructed gene tree than the gene trees simulated under the model of population fragmentation from a single ancestral population, over a number of effective population sizes (Figure 5A). This suggests that either ancestral populations were very large and that in part, incomplete lineage sorting precludes us from inferring population history from 16S mtDNA sequences. Alternatively, historical fragmentation with subsequent secondary contact could also explain some or all of the genetic pattern observed.

### Relation of morphology and call to genealogical patterns

A simple, visual comparison of the patterns of differentiation in morphology and advertisement call (from our CV analyses) to phylogeny suggest no obvious relationship between our two measures of phenotypic distinction and genealogical history. For instance, locales Man and Tab appear little differentiated in call space (Figure 2B), yet all seven individuals sampled from Tab have haplotypes embedded within Clade 1 (haplotypes S, T, U, V), whereas all 10 individuals from Man have haplotypes within Clade 3 (L, M). Although SdN haplotypes are dispersed among divergent Clades 1 (haplotype C) and 3 (A, B) individuals within this population do not exhibit any greater dispersion in call space. Similar discord is evident between morphology and phylogeny. For example, Auk and Obd exhibit the greatest inter-centroid distances on CV axis 1 in Figure 2A, yet haplotypes from both sites are dispersed throughout Clade 1 with no evidence for compartmentalization. Lineages simulated under a population 'stepping stone' model designed to reflect population similarity based on call parameters (Figure 4B) do not reduce the disparity in discord between reconstructed gene and population trees, which remain significantly higher than expected under neutral coalescence.

## Discussion

### Variation and distinction among populations

#### Morphological

*Hyla leucophyllata *does not show sufficient morphological variation to have provoked the naming of subspecies or other taxonomic revision (see Methods). However, upon close examination the species nonetheless demonstrates diagnostic patterns of morphological variation across populations. That significant variation has gone unremarked is perhaps not surprising when the CVA plot for morphology (Figure 2A) is examined in detail. Despite a statistically significant separation in this multivariate space, and indeed on every univariate morphological measure, most populations of *H. leucophyllata *grade into one another in morphological space rather than showing obvious gaps. Most of the variation among populations was in size although there was also a shape component. Elements of size and shape or general appearance feature in most taxonomic descriptions of frogs [18,19]. Thus, in that sense, the variation found here is significant. However, the evolutionary significance of this variation is harder to judge. For example, although size and shape differences among populations might reflect evolutionary divergence in form they could also be due to ontogeny. All individuals were males and almost all were calling and thus presumably sexually mature. However, frogs may undergo some growth-related changes even at this stage [18], so variation among populations might only reflect age class differences and thus sampling bias [20]. Equally problematic would be ecophenotypic variation that is not heritable and thus not indicative of evolved differences among populations. Both factors are hard to rule out without direct experimentation when the taxa studied are allopatric, the characters continuous, and the differences subtle or relatively small.

#### Call

Variation in the advertisement calls of *H. leucophyllata *was both greater and more distinctive than that in morphology. For significantly variable call characters, among-population coefficients of variation were on average more than three times as large as those coefficients for morphological variables. In addition, within-population variation was lower and populations were better separated from one another in call space than in morphological space (Figure 2B). However, inasmuch as calls here serve as an indicator of the MRS, it is insufficient to show variation in structure of calls; rather this variation must also have some bearing on mate choice.

Significant variation among *H. leucophyllata *populations was found in pulse rate and other temporal features like call length. In addition, the difference among populations in dominant frequency was significant if it was not adjusted for body size. All of these features are known to affect mate choice in other hylid frogs [4,21]. Moreover, both pulse rate and dominant frequency are known to function in mate discrimination in another *leucophyllata*- group species, *H. ebraccata *[22]. The number of secondary notes was also significantly different among populations of *H. leucophyllata *and this character is thought important to mate choice in *H. ebraccata *(J. Schwartz pers. comm.). Although there are no direct tests of which characters influence mate choice in *H. leucophyllata*, the tendencies of frogs in general (and a close relationship with *H. ebraccata*, in particular) suggest that at least some of the above characters play a role in mate choice in *H. leucophyllata*.

Significant geographical variation in the call of *H. leucophyllata *is consistent with other studies on frogs (e.g., [6-8,23-26]. However, most of these studies involve calls as corroborating evidence for differences established first on morphological or genetic grounds, or are an explicit attempt to illustrate reproductive character displacement/reinforcement between established species. Few studies examine the potential for call evolution to initiate interpopulation divergence, although recent biogeographic and experimental data have demonstrated that reinforcement may act to drive rapid pre-mating isolation in frogs (Hoskins 27). Regardless, the data presented here for *H. leucophyllata *emphasize the value of considering MRS evolution as a potential cause rather than effect of frog speciation, given that it appears to be under greater selection pressure than either morphology or DNA sequence.

### Phylogenetics

We found striking phylogenetic divisions within *H. leucophyllata *but with little obvious geographic structure. Previous phylogenetic and phylogeographic work on the 30 chromosome clade (including *H. leucophylata *and *H. triangulum*) suggested that neither population spatial proximity nor colour pattern polymorphism corresponds well with mitochondrial phylogeny [28]. Expanded sampling and character evaluation in this study confirms this conclusion. Previous studies of frogs have shown similarly striking phylogenetic divisions within traditionally regarded species. For example, allozymes and immunological techniques have tended to find pronounced genetic divisions within frog species [e.g. [29-31]]. Sequence-based studies of intra-specific variation in frogs also have typically found pronounced genetic divisions within species [32-34]. Here, point estimates of clade divergence times surpass those found in 16S in a temperate hylid [34].

### Relation between character types

The presence of variation in morphology, advertisement call, and DNA sequence permits questions about relationships among these suites of data. The null hypothesis is that evolution in phylogenetic distinction, form, and reproductive isolation should be at least rank correlated during the speciation process; i.e. lineages that are evolutionarily independent will diverge through genetic drift alone given sufficient time. However, we found no such relationship between either morphology or call, and phylogeny. Even pronounced separation between two populations for one suite of characters was not necessarily accompanied by separation for another. A variety of possibilities exist to explain departure from the null expectation of rank correlation. We examine some of these below.

### Single Marker Effects

Despite our finding of strongly supported phylogenetic divisions, this phylogeny is based only on variation of a single marker, the 16S rDNA molecule. Thus, the lack of a concordance between phylogeny and morphological or call divergence found here might then simply reflect a failure to accurately recover historical affinities among populations. However, such correlations can also be absent in studies of frog populations that use a large number of nuclear markers such as allozyme studies [e.g. [31], but see [25]], suggesting the phylogenetic estimate presented here perhaps could accurately reflect the history of connectedness among these populations. Furthermore, mitochondrial DNA is more likely than many single nuclear sequence loci to reflect historical population affinities given the former's smaller effective population size. Given this possibility at least, it is reasonable to explore other explanations for the absence of a correlation between phylogeny and measures of phenotypic distinctiveness.

### Selection on morphology

Insofar as our 16S phylogeny is an accurate depiction of population history, then major clades of *H. leucophyllata *may have been separated from each other for up to several million years [28]. If so, the lack of apparent correspondence between phylogeny and morphology would still not be particularly surprising for frogs. A number of studies have found pronounced genetic differences among frog taxa that are not reflected in morphology [e.g. [29,31,35]]. What morphological differences there are among populations of *H. leucophyllata *could reflect ecophenotypic or ontogenetic effects as noted above. By contrast, deviation from a relation with phylogeny could indicate the action of selection upon morphology and adaptation to local environmental conditions as is suggested in some species-level comparisons [20,36]. Although a correlation of morphological with geographic distance might then be anticipated, the geographic separation among *H. leucophyllata *populations is generally large and, so, even spatially proximate populations could experience substantially different environmental pressures.

Whether morphological differentiation is selected or not, the fact remains that large divergences in DNA sequence relate to seemingly trivial morphological effects in these frogs. Indeed, species of the *leucophyllata*-group are separated on average by more than twice as much genetic distance as are clades of *H*. *leucophyllata *[28] yet, even at the species group level, separation on morphological grounds alone has been historically problematic [37]. Moreover, as noted above, available evidence suggests that these clades show levels of sequence divergence greater than that typical between congeners in other groups like mammals and birds [38]. Why frogs might exhibit conservative morphological evolutionary patterns is not clear. However studies on another group of amphibians with a conserved morphology offers one explanation. Wake et al. [39] suggested that in plethodontid salamanders, an unpredictable environment has selected for a generalized body type coupled with facultative behavioural adjustment. Behavioural flexibility buffers the effect of environmental variation and hence minimizes naturally-selected morphological change over evolutionary time. On this view, genetic changes continue to accrue within lineages but morphological evolution is retarded, leading to decoupling of morphological and molecular evolution [40]. *Hyla leucophyllata *fits the profile described by Wake et al. [39] for a species with an evolutionarily "persistent" morphology. It seems to face and cope with considerable environmental heterogeneity, occupying and breeding in a variety of habitats even within the same locale, from cattle ponds in open fields to closed-canopy forest (Chek, pers. obs). Hence, this hypothesis could explain why apparently deep evolutionary splits in *H. leucophyllata *have not led to obvious and concordant changes in form.

### Selection on body size or calls?

The pattern of call evolution provides some of the most interesting points for consideration. Although call and morphology distance *matrices *are uncorrelated (Table 5), there is a relationship between some aspects of calls and morphology (body size). A few of the 30 call characters measured were correlated to snout-vent-length, but this variation was removed before overall call distance was calculated. Adjustment for body size allowed examination of call variation that is independent of the most obvious influence of morphology. However, call variables correlated to body size are far from unimportant to a consideration of the process of call evolution in *H. leucophyllata*. Of the call variables correlated to body size, the strongest relationship was one with dominant frequency (r^2 ^= 0.61; Table 3): larger males had calls of lower frequency. Females of several frog species are known to prefer larger males as mates and independently to prefer lower dominant frequencies – tendencies true of *H. ebraccata *females [22,41]. As with any correlation, it is difficult to determine whether one factor is driving the other, or whether other factors are causal. Interpopulation variation in body size might be naturally selected, or indeed could be an ecophenotypic response. In both cases, differences in dominant frequency and its reception by females simply would be "dragged along" over time. However, an intriguing possibility is the reversal of this scenario: females could prefer the signal itself with body size evolution a correlated effect. Sexual selection theory provides several models by which direct female preferences and associated male traits could evolve [42]. A potentially generative role for frog calls in morphological evolution is reflective of generally increased research interest in the role of the MRS in initiating speciation, especially with respect to sexual selection [9,11,27]. Moreover, it emphasizes once again the value of investigating multiple character suites.

### Covariation of calls with geography

As with morphology, there was no obvious relation between phylogeny and distance among populations in call (e.g. see Figure 4). Again, this may be due to a failure of the single genetic marker to accurately reflect the history of gene flow among populations; a recent history of gene flow could be obscured by lineage sorting effects. If so, then further study using a suite of nuclear markers could reveal a correlation of phylogeny with call distance; a pattern consistent with classical views that see MRS evolution as a largely pleiotropic effect of divergence in other aspects of the organism [5]. If call divergence among *H. leucophyllata *populations is a result of undetected similarity across nuclear loci, then this may explain the only significant and strong correlation among matrices, that between geography and calls. However, even a study that found a correlation between call divergence and genetic divergence also found a residual correlation of call distance with geographic distance [25], which suggests some other factor that covaries with geographic distance.

One possibility is that locales in closer proximity are more similar in vegetational composition than those that are further apart. Among other effects of vegetation is the degradation of acoustic signals broadcast through it [43]. Frog calls with higher pulse rates are degraded more when transmitted through vegetation than in open habitat formations [44], and there is some evidence that frog species in open habitats have higher pulse rates than those in forest [45]. Thus, slower pulse rates of some *H. leucophyllata *populations might be a selected response of denser vegetation at those locales. However, the lowest pulse rates actually come from populations in swampy open areas (RB, Tab). If current habitat is any guide to the long-term selective *milieu*, then this suggests that geographic variation in vegetation density is not the cause of the observed pattern of call variation among populations.

Another factor that could covary with geographic distance between *H. leucophyllata *populations is the composition of the sympatric frog assemblage. Distribution of frog species in the lowland forest of the Amazon Basin is far from uniform [46]. Of the approximately 200 species found there only about 10% are found throughout the Amazon, with *H. leucophyllata *being one of these [46]. Thus, there is geographic variation in the identity of species co-occurring with *H. leucophyllata*. For example, this species can be sympatric with three other *leucophyllata*-group species in the western Amazon, whereas, in the eastern Amazon, it is, as far as is known, the only member of its group. If, all else being equal, sites closer together are more similar in species composition, then competition among species for "acoustic space" could explain both call divergence among sites and covariation with geographic distance. Call divergence through acoustic competition could occur either as a means of avoiding mis-mating (reproductive character displacement *sensu *47], or of avoiding destructive interference of sound waves, or both. Both mechanisms have been invoked to explain the distribution of call features among species in frog assemblages [reviewed in [48]]. Like its ecological counterpart, hypotheses of acoustic partitioning posit that selection acts to carve out an acoustic niche for the organism such that overlap with neighbours in syntopic soundspace is minimized. Unfortunately, an inventory of the species at each site and recordings of all of their calls is not available to test this hypothesis for *H. leucophyllata*. Nonetheless, there is some evidence for acoustic partitioning in frog assemblages of which *H. leucophyllata *is a part [49].

## Conclusion

*Hyla leucophyllata *exhibits strikingly deep phylogenetic divisions, although the species as currently designated is possibly paraphyletic with *H. triangulum*. Significant differentiation among populations in both external morphology and male advertisement call does not show any obvious relationship to the 16S DNA genealogy implying that, if the latter does mirror the evolutionary history of the species, then in some sense evolution of these different characters is decoupled. A strong relation between call structure and geographic distance may reflect differential acoustic partitioning of *H. leucphyllata *populations within different calling frog assemblages. Some call attributes (notably dominant frequency) show a highly significant relationship to body size raising the possibility that (i) divergence in body size is either ecophenotypic or caused by diversifying selection in different environments, and has resulted in divergence in call frequency, or (ii) selection on call itself [27], mediated by female choice, has produced divergence in body size.

## Methods

### Study species

*Hyla leucophyllata *is a small (20–35 mm) treefrog widely distributed and common throughout the lowland forests of the Amazon Basin [50] and associated habitat. Males usually call above small water bodies from emergent vegetation and may be syntopic with a variety of other species (Chek pers. obs.). *Hyla leucophyllata *is one of six species in the *leucophyllata*-group [50]. Like many such frogs, species group members are characterized by extreme morphological similarity. This leads to the possibility that *H. leucophyllata *is simply a morphotype. However, sympatry of several species [51] with clearly distinct advertisement calls suggests that these taxa are reproductively isolated. Call differences among the species are generally paralleled by large differences at mtDNA loci [28], also supporting the assumption that these taxa are indeed species. *Hyla leucophyllata *previously had not been suggested to comprise more than one species, by any criterion. However, there is some suggestion that at least some lineages within *H. leucophyllata *are implicated in a clade containing *H. triangulum*, and the species as currently designated may be paraphyletic [28]. In addition, noticeable variation in phenotype does occur within *H. leucophyllata*, but is confined to colour-pattern polymorphism. However, particular morphs are not restricted to given populations and allozyme analysis has established that even extreme differences in colour-pattern are not related to species boundaries [52].

### Collections, characters, and measurements

Over three rainy seasons (Nov. 1993 – Feb. 1994; Jan. 1995 – Apr. 1995; Feb. 1996 – Apr. 1996), we collected *H. leucophyllata *(n = 99) from seven sites that span the majority of the species' range. For purposes of phylogeographic analyses we added tissue samples for two additional sites on the Rio Juruá in western Amazonas (see Additional file 1). Inter-site distances ranged from 35 to 1948 km. Figure 1 indicates localities sampled and Additional file 1 lists the disposition of specimens, as well as the approximate latitude and longitude where they were sampled. All individuals collected for this study were males, as judged by calling activity (only males call) or the presence of testes during tissue sampling. Once tissue had been sampled, specimens were fixed in 10% formalin and then stored in 70% ethanol. Sample sizes varied for each character set (Table 1, Additional file). Minimally, most individuals that were measured for calls were also measured for morphology and most of these had associated sequences.

### Morphology and call

Aspects of shape and size are among the most commonly used descriptors in systematic diagnoses of frogs. Seventeen measurements from each individual were collected following Lee and Crump [53] (see Additional file 2 for a list of variables). Measurements were made on preserved specimens to the nearest 0.1 mm using dial calipers. Because colour-pattern is difficult to quantify and its analysis in these frogs may require several thousand individuals [37], colour-pattern was not included as a character.

*Hyla leucophyllata *produces a call composed of a pulsed trill (primary note) that may be followed by one to six shorter secondary notes of similar form (Chek, pers. obs.). Calls were recorded in the field using a Sony WM-D3 Professional Walkman and Electro-Voice (model 635A) microphone. Temperature during recording was always 25–26°C, hence no significant effects of temperature on call characters were expected. Five calls of each individual were digitized at a sampling rate of (10 KHz) using CANARY vers.1.2 (Cornell Bioacoustics). The same program was also used to produce waveforms and sonograms for each call from which variables were measured using built-in software tools. Variables comprised a mix of spectral and temporal properties of calls, including some that are known to influence mate choice in other species (e.g. pulse rate [21]). For each individual, the average of each variable across its five measured calls was used in all analyses. Variable names and definitions are listed in Table 2; Figure 5 shows a typical waveform and sonogram from which call variables were measured.

### Statistical analyses of morphology and call data

For morphological and call variables, means and variances were calculated for raw values of each variable. However, subsequent analyses employed log-transformed values. Because some aspects of frog calls are influenced by body size (e.g. dominant frequency [51,54]), all call variables were regressed against a measure of overall body size (snout-vent-length; SVL). Where a significant relationship between a call variable and SVL was found, the residuals of the regression were used in subsequent analyses. Wherever multiple tests were performed (e.g. all call variables regressed on SVL) a sequential Bonferroni adjustment [55] was made. Most analyses were performed in JMP vers. 3.1.6 (SAS Institute Inc., Cary, North Carolina) and SYSTAT vers. 5.2.1 (Systat Inc., Evanston, Illinois), although some (e.g. Mantel's test) used R-PACKAGE vers. 3.0 [55].

### Amount and distinctiveness of character variation

Multiple characters were measured for calls and morphology to more fully capture the extent and direction of variation in trait space. We tested for differences among populations for each character using a Kruskal-Wallace test. We also used Canonical Variates Analysis (CVA) to examine differences among populations in call and morphology. A technique commonly used in studies of frog morphological and call variation [e.g. [24,26,57]], CVA yields axes that summarize trait variation and produces corrected distances among groups. While the use of CVA with small sample sizes is of some concern, we found that the results were qualitatively similar to those produced by Principal Components Analysis (PCA), a technique with less stringent assumptions. PCA, however, is not recommended for an analysis of geographic variation [58] because it does not allow for the *a priori *consideration of groups (populations).

CVA yielded corrected (Mahalanobis) distances of each individual from each population centroid, including the centroid of its own population. Individual distances from each population to a given centroid were averaged to produce an inter-population distance. This procedure was repeated for all pairs of populations to produce a matrix of pairwise distances. The diagonal of the matrix represents the average distance of individuals from their own population centroid. The ability of each CVA to discriminate diagnostic variation among populations was assessed by its success at correctly classifying individuals to the population from which they originally were drawn.

### Relation of morphology, call and geography

Mantel's test was used to calculate correlations between all distance matrices (call, morphological, a matrix of straight-line geographical geographic distances between sites) [59]. One thousand permutation replicates were performed for each test. Distances within matrices were first ranked because the principal question concerned whether the relative magnitudes of variation were correlated. However, the use of unranked matrices did not change any conclusions. Scales of measurement among character sets were made comparable by dividing each distance matrix by its maximum value; thus, in rescaled matrices the maximum value was one.

### Phylogeny

For general details on tissue collection and amplification and sequencing of a fragment of the mitochondrial 16S rRNA gene see [28] Chek et al. (2001). DNA sequence data from the 16S rRNA gene were used to estimate the genealogical history of sampled populations. Gaps were treated as missing data for all analyses. Point estimates of divergence among major clades was estimated following the moment method [60], which corrects for the ancestral portion of within-clade diversity (*p*_net _= *p*_AB _- 0.5 [*p*_A_+*p*_B_]).).

Given the aforementioned possibility of paraphyly of *H. leucophyllata*, we included sequence from both it and *H. triangulum*. We approached inferring the genealogical relationships of *H. leucophyllata *using both maximum likelihood (ML) and Bayesian approaches. We included *Hyla elegans *as an outgroup, as it lies unequivocally outside of our *leucophyllata*/*triangulum *ingroup [28]. For ML, we used MODELTEST vers. 3.06, [61] to select the best model of evolution (GTR + I + G; with proportion of invariant sites (I) = 0.371, γ shape parameter = 0.491). We conducted ML using an exhaustive search and 'as-is' sequence addition, and evaluated support for resulting topologies using 100 nonparametric bootstrap pseudoreplicates with PAUP* vers. 4.10 [62]. We also ran two simultaneous Bayesian analyses (MR.BAYES vers.3.1.1; [63], each beginning with random starting trees, with Metropolis-coupled MCMC using four incrementally heated Markov chains sampled every 100 generations. We estimated stationarity of the Markov chain by plotting the sampled log likelihood scores versus generation time. Analyses were run for 1.0 × 10^6 ^generations until the average standard deviation of split frequencies was less than 0.01. Potential scale reduction factors (PSRFs; see [64] for all estimated parameters was close to 1.00 and estimated effective sample sizes (using the program TRACER vers. 1.2.1 [65]) for all parameters were all > 100, both suggesting that we had adequately sampled the posterior distribution of trees. Trees generated before the burn of 250000 generations were discarded and we used the remaining trees to generate 50% majority rule consensus trees.

### Coalescent simulations of molecular evolution

We conducted coalescent simulations of molecular evolution and applied a gene-tree/population-tree approach [66] to estimate whether the 24 haplotypes in the reconstructed 16S gene tree was concordant with two population models representing 1) divergence from a single ancestral population, and 2) population history as predicted by the significant correlation between call variables and geographic distance (see results). All simulations and measures of discordance were done in Mesquite version 1.05 [67]. Reconstructed haplotypes were contained within populations under both population models listed above. We used *s *[68] to measure the discord between the gene tree and its subdivision into populations (treating later as categorical variables), with *s *used to infer time since divergence assuming no gene flow. For population divergence from a single ancestral population we apply a star model; appropriate because there we found no evidence of genetic isolation by distance. The second population model represents a stepping stone model reflecting the strong relationship between population similarity in call variables and geographic proximity of populations. Specifically, a distance matrix based on the CVA of measured call parameters was used to construct a population tree, which was then used to represent population history that follows a stepping stone model of divergence.

Simulated gene-trees were created using the Genesis package of Mesquite vers. 1.05. For each simulation we created 500 gene trees for each of three temporal scenarios: Ne = 5000, 10,000, and 40,000, where Ne represents time since populations splitting measured in generations [67]. Given that small sample sizes make estimating theta from a single marker problematic, and the difficulty in obtaining accurate mutation rate estimates for 16S in treefrogs, we chose to simplify the model by assuming each historical population had similar effective population sizes. Within Mesquite, this is equivalent to holding the branch widths of the population tree equal across populations (branch widths equal 1). Parameters were modelled using empirically derived nucleotide frequencies, proportion of invariant characters, gamma shape parameter, and six-parameter rate matrix model (see above). A scaling factor was selected through preliminary runs that provided pairwise sequence divergence rates similar to our observed 16S data (scaling factor = 2.0 × 10^-6^) [67]. The 500 simulated gene-trees for each generation time provided an expected distribution of random coalescent gene-trees from which to compare observed values of *s *under the two population hypotheses.

## Authors' contributions

This manuscript evolved from a portion of AAC's PhD thesis, under the supervision of JPB. The field measurements and samples were collected by AAC and JPB. SCL JDA and AAC conducted the majority of the laboratory work, statistical analyses and writing of the manuscript. JPB and PTB contributed ideas, and financial assistance throughout the project. All authors commented on and approved the final draft of this manuscript.

## Supplementary Material

Additional File 1 **Voucher specimens and locality information. **All individuals were measured for morphological variables. Individuals in bold type were measured for call variables. Asterisks indicate individuals that were sequenced. Genebank accession numbers are indicated in square brackets. All individuals have been deposited with the Museum of Zoology of the University of São Paulo (MZUSP) or the Instituto Nacional de Pesquisas da Amazonia (INPA). Click here for file

Additional File 2 **Summary statistics and univariate tests for morphology. **Population means (above) and standard deviations (below) for 17 morphometric measurements of *Hyla leucophyllata *for all individuals from each population Locations are shown in Figure 1. All measurements are in mm. Kruskal-Wallis tests with α levels adjusted by the sequential Bonferroni method showed that all variables differed significantly across populations (p < < 0.05). Click here for file

Additional File 3 **Variable loadings and eigenvalues for morphology CVA. **Correlation coefficients of each morphological variable with canonical axes (loadings) and associated eigenvalues for each axis. For abbreviations of morphological variables see Additional file 2. Click here for file

Additional File 4 **Summary statistics and univariate tests for call variables. **Population summaries for 30 call measurements of *Hyla leucophyllata *for all individuals from each population. Localities are shown in Figure 1; variable descriptions in Table 2. Variables that differed significantly by Kruskal-Wallis tests (p < 0.05) after sequential Bonferroni adjustment of alpha level are indicated by an asterisk (*). A t-bar (†) indicates that the residuals on the snout-vent-length were used in the Kruskal-Wallis test and CVA (see Table 3). Click here for file

Additional File 5 **Variable loadings and eigenvalues for call CVA. **Correlation coefficients of call variables with canonical axes (loadings) and associated eigenvalues for each axis of the CVA. Click here for file
