# Supplementary material for: Multi-character perspectives on the evolution of intraspecific differentiation in a neotropical hylid frog
Source: BMC Evol Biol. 2006 Mar 15;6:23. doi: 10.1186/1471-2148-6-23 (PMC1434785; doi:10.1186/1471-2148-6-23)
Supplement: Additional File 1 — Voucher specimens and locality information. All individuals were measured for morphological variables. Individuals in bold type were measured for call variables. Asterisks indicate individuals that were sequenced. Genebank accession numbers are indicated in square brackets. All individuals have been deposited with the Museum of Zoology of the University of São Paulo (MZUSP) or the Instituto Nacional de Pesquisas da Amazonia (INPA). [file 1471-2148-6-23-S1.pdf]

Additional file 1: Voucher specimens and locality information. All individuals were measured for morphological variables. Individuals in bold type were measured for call variables. Asterisks indicate individuals that were sequenced. Genebank accession numbers are indicated in square brackets at their first appearance. All individuals have been deposited with the Museum of Zoology of the University of São Paulo (MZUSP) or the Instituto Nacional de Pesquisas da Amazonia (INPA).

Hyla leucophyllata

AdC: Alter do Chão (2°32' S, 54°58' W) Field Numbers: **95161*** [haplotype1 DQ393416], **95162*** [haplotype7 DQ393422], **95163*** [haplotype4 DQ393419], **95164*** [haplotype4], **95165*** [haplotype4], **95166*** [haplotype7], **95167*** [haplotype4], 95168* [haplotype4], 95169* [haplotype4].

Aukre: approx. 200km west of Redenção, Para, Brasil. (7°39' S, 51°21' W) Field Numbers: **93041*** [haplotype4], **93042, 93043*** [haplotype3 DQ393418], **93044*** [haplotype6 DQ393421], **93045*** (= MZUSP 70159) [haplotype3], **93046*** (= MZUSP 70160) [haplotype3], **93047*** [haplotype3], **93048*** [haplotype3], **93049*** [haplotype5 DQ393420], **93050*** [haplotype3], 93051, 94014, 94015, 94016, 94017, 94018, 94019, 94020, 94021* [haplotype3], 94022, 94023, 94024, 94137, 94138, 94159, 94175, 94176.

Man: approx. 100 km north of Manaus, Amazonas, Brasil (3°08' S, 60° 01' W) Field Numbers: 95193* [haplotype13 DQ393428], 95194* [haplotype13], 95195* [haplotype13], 95196* [haplotype13], **95230*** [haplotype13], **95231*** [haplotype13 DQ393428] **95232*** [haplotype12 DQ393427], 95233* [haplotype13], 95234* [haplotype13], 95235* [haplotype13], 95236.

Obd: Obidos, Pará, Brasil (1°55' S, 55°31' W) Field Numbers: **95172*** [haplotype11 DQ393426], **95173, 95174*** [haplotype3], **95175*** [haplotype8 DQ393423], **95176*** [haplotype9 DQ393424], **95177*** [haplotype3], **95178*** [haplotype10 DQ393425], **95179*** [haplotype4], 95180, 95181, 95182* [haplotype11], **95183*** [haplotype11], 95185* [haplotype11], **95186*** [haplotype11], 95187* [haplotype11], **95191**, **95192**.

RB: Rio Branco, Acre, Brasil. (9° 58' S, 67°48' W). Field Numbers: **95252*** [haplotype1], **95253*** [haplotype17 DQ393432], **95254*** [haplotype18 DQ393433].

SdN: Serra do Navio, Amapá, Brasil (0°59' N, 52°03' W) Field Numbers: 95122, 95123, 95127, 95128, 95140, 95141, 95142* [haplotype1], **95143*** [Haplotype2 DQ393417], 95144* [haplotype1], 95145* [haplotype1], 95146* [haplotype1], 95147* [haplotype1], 95148, 95149, 95150, **95151*** [haplotype1], **95152*** [haplotype1], **95153*** [haplotype1], **95154*** [haplotype3], 95155, **95156*** [haplotype1], **95157*** [haplotype1], **95158**, 95159, 95160.

Tab: Tabatinga, Amazonas, Brasil (4°15' S, 69° 57' W) Field Numbers: **96009, 96018*** [haplotype20 DQ393435], **96019,** 96020* [haplotype21 DQ393436] 96022* [haplotype20], **96023*** [haplotype14 DQ393429], 96034* [haplotype19 DQ393434], **96035, 96038, 96056*** [AF308094 now haplotype21], **96057*** [haplotype20].

Por: Igarapé Porongaba, Acre, Brasil (8°40' S, 72° 47' W) Field Numbers (Claude Gascon): 4273* [haplotype16 DQ393431].

NV: Nova Vida, Acre, Brasil (8°22' S, 72° 49' W) Field Numbers (Claude Gascon): 4483* [haplotype15 DQ393430].

Hyla triangulum

Sacado, Amazonas, Brasil (6°45' S, 70° 51' W) Field Numbers (Claude Gascon): 2976* [haplotype23 DQ393438].

Tabatinga, Amazonas, Brasil (4°15' S, 69° 57' W) Field Numbers: 93042* [haplotype 22 DQ393437], 96053* [haplotype 24 DQ393439].

Hyla elegans

Teresopolis, Rio de Janeiro, Brasil (22°25' S, 43° 0' W) Field Numbers: 95029* [AF308102], 95033* [AF308103].
