# Supplementary material for: Multi-character perspectives on the evolution of intraspecific differentiation in a neotropical hylid frog
Source: BMC Evol Biol. 2006 Mar 15;6:23. doi: 10.1186/1471-2148-6-23 (PMC1434785; doi:10.1186/1471-2148-6-23)
Supplement: Additional File 2 — Summary statistics and univariate tests for morphology. Population means (above) and standard deviations (below) for 17 morphometric measurements of Hyla leucophyllata for all individuals from each population Locations are shown in Figure 1. All measurements are in mm. Kruskal-Wallis tests with α levels adjusted by the sequential Bonferroni method showed that all variables differed significantly across populations (p < < 0.05). [file 1471-2148-6-23-S2.pdf]

Additional file 2: Population means (above) and standard deviations (below) for 17 morphometric measurements of *Hyla leucophyllata* for all individuals from each population Locations are shown in Figure 1. All measurements are in mm. Kruskal-Wallis tests with α levels adjusted by the sequential Bonferroni method showed that all variables differed significantly across populations ($p << 0.05$).

**Pop**	**SVL**	**AGL**	**FEL**	**TBL**	**FOL**	**MET**	**FDD**	**RUL**	**HAL**
AdC	30.3	15.4	14.7	15.9	13.6	8.9	1.5	6.3	8.7
	(0.8)	(1.0)	(0.6)	(0.6)	(0.7)	(0.4)	(0.2)	(0.3)	(0.6)
Aukre	31.0	16.1	15.6	15.9	14.1	8.6	1.4	6.4	9.0
	(1.1)	(1.2)	(0.6)	(0.6)	(0.7)	(0.5)	(0.2)	(0.4)	(0.4)
Man	30.8	14.5	15.1	16.4	14.3	9.1	1.3	6.3	9.2
	(1.1)	(1.3)	(0.8)	(0.7)	(0.5)	(0.3)	(0.2)	(0.4)	(0.5)
Obd	24.9	12.3	11.4	12.6	10.8	7.2	1.3	5.1	6.9
	(0.9)	(0.5)	(0.7)	(0.6)	(0.5)	(0.4)	(0.1)	(0.3)	(0.4)
RB	30.6	16.2	14.9	15.8	14.4	9.2	1.5	6.2	9.3
	(0.8)	(0.8)	(0.5)	(0.2)	(0.2)	(0.0)	(0.2)	(0.4)	(0.5)
SdN	28.3	15.0	14.1	15.0	13.0	8.2	1.4	5.8	8.2
	(2.4)	(1.6)	(1.2)	(1.4)	(1.0)	(0.7)	(0.2)	(0.6)	(0.8)
Tab	26.9	14.3	12.7	14.0	11.6	8.0	1.2	5.7	7.6
	(1.1)	(0.7)	(1.2)	(1.0)	(0.7)	(0.5)	(0.2)	(0.3)	(0.5)
**p**	<0.0001	<0.0001	<0.0001	<0.0001	<0.0001	<0.0001	<0.0001	<0.0001	<0.0001
$\bar{X}$	29.0	14.8	14.1	15.1	13.1	8.5	1.4	6.0	8.4
**SD**	2.2	1.2	1.4	1.3	1.3	0.7	0.1	0.4	0.8
**CV**	7.5	8.3	10.0	8.3	9.9	7.9	8.3	7.3	9.8

Additional file 2 continued:

Pop	HDD	HEL	HEW	SNL	SND	IND	IOD	EYD
AdC	1.7	10.0	9.9	2.8	2.2	2.1	5.9	3.7
	(0.2)	(0.4)	(0.3)	(0.2)	(0.1)	(0.2)	(0.3)	(0.3)
Aukre	1.5	9.9	9.4	2.7	2.3	2.5	6.1	3.8
	(0.2)	(0.4)	(0.4)	(0.2)	(0.2)	(0.2)	(0.3)	(0.2)
Man	1.6	10.0	9.9	2.8	2.2	2.3	6.4	4.0
	(0.2)	(0.4)	(0.5)	(0.2)	(0.1)	(0.3)	(0.4)	(0.3)
Obd	1.3	8.3	8.2	2.4	1.9	2.0	5.1	3.2
	(0.1)	(0.4)	(0.3)	(0.1)	(0.2)	(0.2)	(0.3)	(0.3)
RB	1.6	10.3	9.7	2.8	2.4	2.3	6.0	3.9
	(0.1)	(0.3)	(0.3)	(0.2)	(0.1)	(0.3)	(0.5)	(0.2)
SdN	1.5	9.3	9.0	2.8	2.2	2.2	6.0	3.5
	(0.2)	(0.7)	(0.6)	(0.4)	(0.2)	(0.3)	(0.5)	(0.2)
Tab	1.2	8.7	8.3	2.6	2.2	2.1	5.6	3.6
	(0.1)	(0.5)	(0.5)	(0.1)	(0.2)	(0.2)	(0.7)	(0.3)
**p**	<0.0001	<0.0001	<0.0001	<0.0001	<0.0001	<0.0001	<0.0001	<0.0001
$\bar{\mathbf{x}}$	1.5	9.5	9.2	2.7	2.2	2.2	5.9	3.7
**SD**	0.2	0.7	0.7	0.1	0.2	0.2	0.4	0.2
**CV**	11.1	7.3	7.3	5.4	7.3	7.0	6.4	6.5

Snout vent length (SVL); axilla-groin length (AGL); femur length (FEL); tibia length (TBL); foot length (FOL); metatarsal length (MET); foot disc diameter (FDD); radioulna length (RUL); hand length (HAL); hand disc diameter (HDD); head length (HEL); head width (HEW); snout length (SNL); snout depth (SND); internarial distance (IND); interorbital distance (IOL); eye diameter (EYD) as defined by Lee and Crump (1981). See Figure 1 for population codes.
