# Supplementary material for: Multi-character perspectives on the evolution of intraspecific differentiation in a neotropical hylid frog
Source: BMC Evol Biol. 2006 Mar 15;6:23. doi: 10.1186/1471-2148-6-23 (PMC1434785; doi:10.1186/1471-2148-6-23)
Supplement: Additional File 3 — Variable loadings and eigenvalues for morphology CVA. Correlation coefficients of each morphological variable with canonical axes (loadings) and associated eigenvalues for each axis. For abbreviations of morphological variables see Additional file 2. [file 1471-2148-6-23-S3.pdf]

Additional file 3: Correlation coefficients of each morphological variable with canonical axes (loadings) and associated eigenvalues for each axis. For abbreviations of morphological variables see Additional file 2.

Variable	CV I	CV II	CV III	CV IV	CV V	CV VI
SVL	0.629	-0.336	-0.084	-0.207	0.054	-0.296
FEL	0.710	-0.280	-0.315	-0.119	-0.033	-0.322
TBL	0.570	-0.411	-0.338	-0.276	0.163	-0.323
FOL	0.711	-0.467	-0.278	-0.082	0.067	0.138
MET	0.414	-0.428	-0.139	-0.425	0.220	0.004
FDD	0.087	-0.195	-0.060	-0.036	-0.369	0.067
HEL	0.494	-0.390	-0.057	-0.210	-0.077	-0.101
HEW	0.438	-0.619	-0.120	0.006	0.031	-0.179
EYD	0.338	-0.208	0.108	-0.295	0.354	0.035
IOD	0.353	-0.187	-0.400	-0.036	0.219	-0.072
IND	0.307	0.069	-0.030	0.124	0.003	0.138
SNL	0.194	-0.167	-0.421	-0.150	-0.055	-0.150
HDD	0.180	-0.350	-0.082	-0.051	-0.209	-0.151
RUL	0.461	-0.194	-0.074	-0.311	0.097	-0.456
HAL	0.601	-0.343	-0.120	-0.222	0.144	0.064
AGL	0.448	-0.034	-0.282	-0.380	-0.384	-0.200
SND	0.349	-0.016	-0.117	-0.318	-0.071	0.113
**Eigenval.**	6.1694	2.2089	1.0653	0.703	0.5707	0.1545
**% var.**	56.7	20.3	9.8	6.4	5.2	1.4
