# Supplementary material for: Multi-character perspectives on the evolution of intraspecific differentiation in a neotropical hylid frog
Source: BMC Evol Biol. 2006 Mar 15;6:23. doi: 10.1186/1471-2148-6-23 (PMC1434785; doi:10.1186/1471-2148-6-23)
Supplement: Additional File 4 — Summary statistics and univariate tests for call variables. Population summaries for 30 call measurements of Hyla leucophyllata for all individuals from each population. Localities are shown in Figure 1; variable descriptions in Table 2. Variables that differed significantly by Kruskal-Wallis tests (p < 0.05) after sequential Bonferroni adjustment of alpha level are indicated by an asterisk (*). A t-bar (†) indicates that the residuals on the snout-vent-length were used in the Kruskal-Wallis test and CVA (see Table 3). [file 1471-2148-6-23-S4.pdf]

Additional file 4: Population summaries for 30 call measurements of *Hyla leucophyllata* for all individuals from each population. Localities are shown in Figure 1; variable descriptions in Table 2. Variables that differed significantly by Kruskal-Wallis tests ($p<0.05$) after sequential Bonferroni adjustment of alpha level are indicated by an asterisk (*). A t-bar (†) indicates that the residuals on the snout-vent-length were used in the Kruskal-Wallis test and CVA (see Table 3).

**Pop**	**1° dom freq. (Hz)**	**1° FM range**	**1° FM sweep**	**2° dom freq (Hz)**	**2° FM range**	**2° FM sweep**	**call length (s)**	**int-note int. (s)**	**1° note leng (s)**
AdC	2553 (94)	75 (61)	1275 (1953)	2552 (88)	-26 (43)	-486 (785)	0.23 (0.020)	0.039 (0.006)	0.138 (0.039)
Aukre	2559 (77)	49 (34)	518 (356)	2591 (92)	-8 (9)	-177 (231)	0.278 (0.061)	0.041 (0.005)	0.112 (0.009)
Man	2519 (29)	91 (36)	1086 (352)	2527 (14)	-11 (18)	-295 (295)	0.205 (0.036)	0.01 (0.013)	0.131 (0.023)
Obd	3030 (106)	146 (86)	1135 (657)	3045 (115)	4 (16)	121 (303)	0.249 (0.032)	0.034 (0.009)	0.168 (0.017)
RB	2647 (128)	87 (90)	462 (427)	2554 (92)	0 (0)	0 (0)	0.221 (0.017)	0.007 (0.003)	0.206 (0.027)
SdN	2872 (114)	159 (243)	1499 (1658)	2865 (77)	3 (40)	-49 (732)	0.178 (0.019)	0.04 (0.004)	0.112 (0.034)
Tab	2670 (173)	121 (50)	812 (360)	2714 (158)	-31 (56)	-1042 (2160)	0.244 (0.026)	0.047 (0.012)	0.198 (0.015)
**p**	0.0154†	0.0751	0.2322	0.0433†	0.2613	0.1712	0.0007*	0.0022*	0.0001*
$\bar{\mathbf{x}}$	2693	104	970	2693	12	-275	0.229	0.031	0.152
**SD**	190	39	388	196	12	393	0.032	0.016	0.039
**CV**	7	38	40	7	101	143	14.082	51.716	25.624

Additional file 4 continued:

Pop	1° note +int (s)	1° rise time (s)	1° note shape	#1° pulses	1° pulse leng (s)	1° pulse + int (s)	1° pulse rise (s)	1° pulse shape	1° pulse duty
AdC	0.176 (0.038)	0.094 (0.035)	0.669 (0.072)	17.007 (1.488)	0.006 (0.000)	0.008 (0.000)	0.002 (0.001)	0.301 (0.127)	0.794 (0.048)
Aukre	0.153 (0.012)	0.079 (0.014)	0.707 (0.098)	14.825 (0.774)	0.006 (0.001)	0.008 (0.000)	0.001 (0.000)	0.226 (0.038)	0.806 (0.082)
Man	0.14 (0.012)	0.074 (0.014)	0.566 (0.031)	18.6 (1.058)	0.005 (0.000)	0.006 (0.000)	0.002 (0.001)	0.354 (0.148)	0.838 (0.042)
Obd	0.2 (0.014)	0.108 (0.015)	0.648 (0.108)	21.675 (2.290)	0.007 (0.001)	0.008 (0.001)	0.002 (0.001)	0.251 (0.055)	0.812 (0.089)
RB	0.172 (0.011)	0.134 (0.054)	0.645 (0.167)	18.933 (3.029)	0.009 (0.001)	0.011 (0.000)	0.002 (0.000)	0.178 (0.035)	0.785 (0.031)
SdN	0.141 (0.011)	0.067 (0.025)	0.591 (0.122)	15.363 (5.005)	0.006 (0.001)	0.008 (0.000)	0.001 (0.000)	0.232 (0.061)	0.78 (0.077)
Tab	0.24 (0.020)	0.125 (0.021)	0.635 (0.108)	22.65 (1.559)	0.007 (0.001)	0.009 (0.000)	0.001 (0.000)	0.218 (0.039)	0.774 (0.098)
**p**	0.0001*†	0.0001*	0.2941	0.0002*†	0.0085	0.0001*	0.8565	0.2281	0.8428
$\bar{\mathbf{x}}$	0.175	0.097	0.637	18.436	0.007	0.008	0.002	0.251	0.798
**SD**	0.036	0.026	0.047	2.974	0.001	0.002	0	0.059	0.022
**CV**	20.494	26.549	7.391	16.129	18.389	20.123	11.683	23.271	2.764

Additional file 4 continued:

Pop	1° pulse rate (Hz)	2° note leng (s)	2° note rise (s)	2° note shape	# 2° notes	# 2° pulses	2° pulse leng (s)	2° pulse +inter (s)	2° pulse rise (s)
AdC	131.467 (12.349)	0.035 (0.005)	0.018 (0.004)	0.519 (0.065)	1.255 (0.216)	5.769 (0.683)	0.005 (0.001)	0.006 (0.001)	0.002 (0.001)
Aukre	133.014 (8.316)	0.034 (0.005)	0.017 (0.004)	0.506 (0.093)	2.225 (0.767)	5.05 (0.593)	0.005 (0.001)	0.006 (0.001)	0.001 (0.000)
Man	145.458 (22.528)	0.066 (0.028)	0.016 (0.009)	0.249 (0.085)	1 (0.000)	9.578 (2.989)	0.005 (0.001)	0.006 (0.001)	0.002 (0.001)
Obd	128.992 (5.805)	0.042 (0.012)	0.012 (0.003)	0.301 (0.094)	1.056 (0.210)	6.226 (1.805)	0.006 (0.001)	0.007 (0.001)	0.001 (0.001)
RB	91.857 (3.092)	0.052 (0.017)	0.016 (0.010)	0.29 (0.091)	0.2 (0.200)	5 (1.414)	0.008 (0.002)	0.009 (0.002)	0.002 (0.001)
SdN	136.193 (5.133)	0.03 (0.005)	0.014 (0.003)	0.487 (0.145)	0.925 (0.399)	5.179 (0.881)	0.005 (0.000)	0.006 (0.001)	0.002 (0.000)
Tab	114.797 (6.847)	0.036 (0.010)	0.01 (0.004)	0.27 (0.071)	0.55 (0.256)	4.969 (1.105)	0.006 (0.001)	0.008 (0.002)	0.001 (0.001)
**p**	0.0001*	0.0341	0.3691†	0.0001*	0.0001*	0.053	0.0129	0.009	0.7521
**$\bar{x}$**	125.968	0.042	0.015	0.375	1.03	5.967	0.006	0.007	0.002
**SD**	17.615	0.013	0.003	0.122	0.634	1.66	0.001	0.001	0.001
**CV**	13.984	30.39	19.541	32.711	61.534	27.823	17.693	16.349	15.517

Additional file 4 continued:

Pop	2° pulse shape	2° pulse duty	2° pulse rate (Hz)
AdC	0.337 (0.094)	0.823 (0.085)	165.921 (21.636)
Aukre	0.246 (0.055)	0.842 (0.052)	152.221 (15.781)
Man	0.365 (0.100)	0.853 (0.071)	153.543 (32.951)
Obd	0.244 (0.120)	0.821 (0.062)	155.522 (12.795)
RB	0.195 (0.039)	0.858 (0.001)	97.271 (3.860)
SdN	0.342 (0.104)	0.848 (0.061)	175.911 (15.310)
Tab	0.227 (0.133)	0.871 (0.045)	141.366 (15.656)
**p**	0.0808	0.6755	0.0057
$\bar{x}$	0.279	0.845	148.822
**SD**	0.067	0.018	25.226
**CV**	23.988	2.158	16.95
