# Supplementary material for: Multi-character perspectives on the evolution of intraspecific differentiation in a neotropical hylid frog
Source: BMC Evol Biol. 2006 Mar 15;6:23. doi: 10.1186/1471-2148-6-23 (PMC1434785; doi:10.1186/1471-2148-6-23)
Supplement: Additional File 5 — Variable loadings and eigenvalues for call CVA. Correlation coefficients of call variables with canonical axes (loadings) and associated eigenvalues for each axis of the CVA. [file 1471-2148-6-23-S5.pdf]

Additional file 5: Correlation coefficients of call variables with canonical axes (loadings) and associated eigenvalues for each axis of the CVA.

Variable	CV I	CV II	CV III	CV IV	CV V	CV VI
1° pulse rate	0.131	-0.012	-0.037	-0.236	0.081	0.057
1° pulse length	-0.070	0.037	0.022	0.207	-0.094	-0.109
1° pulse + inter	-0.153	0.065	0.082	0.510	-0.206	-0.101
1° rise time	-0.073	-0.028	0.149	0.001	0.090	-0.219
1° pulse rise	-0.004	0.000	-0.018	0.003	0.160	-0.101
1° note length	-0.154	-0.005	0.211	-0.020	0.187	-0.224
1° pulse shape	0.013	-0.021	-0.037	-0.087	0.223	-0.064
1° pulse duty	0.002	0.007	-0.025	-0.044	0.009	-0.079
1° note shape	0.021	-0.039	0.028	0.021	-0.063	-0.182
1° dom freq.	0.031	0.087	-0.045	0.003	-0.050	-0.060
1° FM range	-0.014	0.034	0.094	-0.091	0.083	-0.056
1° FM sweep	0.012	0.016	0.022	-0.033	0.133	0.007
#1° pulses	-0.131	-0.131	0.099	-0.149	0.308	-0.300
# 2° notes	0.157	-0.084	-0.107	-0.056	-0.353	-0.501
2° pulse rise	-0.001	-0.009	-0.031	-0.015	0.096	0.126
2° pulse duty	-0.018	-0.017	-0.001	0.005	-0.060	0.150
2° note shape	0.098	-0.039	-0.079	0.205	-0.053	-0.008
1° note +inter	-0.092	-0.126	0.155	0.083	0.105	-0.074
# 2° pulses	0.001	0.009	-0.058	-0.192	0.212	-0.068
2° pulse rate	0.108	0.014	0.044	-0.063	0.126	0.176
2° pulse shape	0.033	0.001	-0.050	-0.014	0.151	0.151
2° pulse +inter	-0.079	0.004	0.037	0.023	-0.111	-0.071
2° pulse length	-0.077	-0.003	0.031	0.018	-0.114	-0.011
2° note length	-0.050	0.008	-0.078	-0.181	0.147	-0.114
inter-note int.	0.061	-0.072	0.214	0.142	-0.187	0.170
call length	-0.008	-0.077	0.063	-0.034	-0.193	-0.477
2° note rise	0.029	0.015	-0.023	0.019	0.057	-0.157
2° dom freq	0.033	0.073	-0.034	-0.005	-0.103	-0.018
2° FM range	0.011	0.058	-0.021	-0.032	-0.076	-0.068
2° FM sweep	0.017	0.042	-0.035	-0.018	-0.022	-0.123
**Eigenvalues**	92.464	40.027	23.767	10.084	3.682	2.258
**% variance**	53.7	23.2	13.8	5.8	2.1	1.3
